# Effects of MitraClip on cognitive and psychological function in heart failure patients: the sicker the better

**DOI:** 10.1186/s40001-019-0371-z

**Published:** 2019-02-22

**Authors:** Valentin Terhoeven, Christoph Nikendei, Anna Cranz, Matthias Weisbrod, Nicolas Geis, Philip W. Raake, Hugo A. Katus, Wolfgang Herzog, Hans-Christoph Friederich, Jobst-Hendrik Schultz, Sven T. Pleger

**Affiliations:** 1 Centre for Psychosocial Medicine, Department of General Internal Medicine and Psychosomatics, University Hospital Heidelberg, Thibautstrasse 4, 69115 Heidelberg, Germany; 2 Center of Psychosocial Medicine, Department of General Psychiatry, University of Heidelberg, Voßstrasse 2, 69115 Heidelberg, Germany; 3 Department of Psychiatry and Psychotherapy, SRH Klinikum Karlsbad-Langensteinbach, Guttmannstrasse 1, 76307 Karlsbad-Langensteinbach, Germany; 4 Department of Cardiology, Angiology, Pneumology, Medical Hospital, University of Heidelberg, Im Neuenheimer Feld 410, 69120 Heidelberg, Germany

**Keywords:** Chronic heart failure, MitraClip intervention, Cognitive performance, Memory, Executive function, Depression, Quality of life

## Abstract

**Purpose:**

Cognitive impairment and reduced quality of life is a common condition in patients with heart failure (HF). Percutaneous mitral valve repair using (PMVR) MitraClip (MC) has emerged as a promising interventional tool, reducing all-cause mortality and hospitalization as well as increasing cognitive functioning and quality of life. However, the benefit of HF patients with severely depressed cognitive functioning remains unknown.

**Methods:**

We assessed cognitive functioning (figural memory—FGT, executive function—TOL, TMT B), psychosocial functioning (depression—PHQ-9, quality of life—SF36), and clinical parameters (echocardiography, 6-min walk test distance, and cardiac biomarkers) 1 day before (*t*0) and 6 weeks after (*t*1) MC intervention in HF patients (*n *= 40). First, paired sample *t* tests were conducted to uncover improvements in cognitive functioning post-MC intervention. Second, the COGBAT Norm-sample, a representative age-matched healthy sample, was used to compare participants’ individual scores. Third, bivariate linear regressions were calculated for all key predictors of the detected improvements in cognitive functioning post-MC intervention (*t*1–*t*0).

**Results:**

Following the MC intervention, we found significant improvements in figural memory, executive functioning, and psychosocial functioning. Most of the patients with depressed executive functioning before the MC intervention showed post-intervention test scores within the normal range (> 50th percentile; *t*0 22.5% vs. *t*1 60%) as compared to the normative COGBAT sample. Regression analyses revealed that lower baseline scores in planning ability before the MC intervention (*t*0) were associated with greater planning ability (TOL; *B *= − 0.78, 95% CI − 1.04 to − 0.53), figural memory (FGT; *B *= − 0.26, 95% CI − 0.44 to − 0.07), and cognitive flexibility (TMT B; *B *= − 0.36, 95% CI − 0.50 to − 0.23) improvement post-MC intervention (*t*1–*t*0). Psychosocial functioning and age were not associated with these improvements.

**Conclusions:**

Patients with depressed executive functioning showed the greatest benefit from the MC intervention regarding cognitive functioning. Age and psychological functioning seem less important for cognitive performance improvements post-MC intervention. Hence, severely depressed cognitive functioning in patients is not a contraindication for PMVR using MitraClip.

## Introduction

Systolic heart failure (HF) is a major cause of morbidity and mortality worldwide [1, 2]. The disease is incurable and progressive and exhibits a 5-year mortality of ~ 50% [1, 2]. For more than 10 years, it has been known that the occurrence of severe mitral valve regurgitation (MR) aggravates cardiac remodeling, exacerbates clinical symptoms and is an independent negative predictor of mortality in HF [3]. Moreover, HF patients commonly display burdensome psychosocial as well as cognitive symptoms, including impaired quality of life, concentration and alertness [2]. Recent studies, applying elaborate methodological paradigms to assess cognitive functioning, have confirmed an association between the severity of HF and cognitive impairment, particularly for the cognitive domains of memory and executive functioning [4, 5].

Percutaneous mitral valve (MV) repair (PMVR) using MitraClip is an established technique to treat severe mitral valve regurgitation (MR) in high-risk surgical patients after interdisciplinary discussion [1, 2, 5–11]. PMVR was recently shown to reduce all-cause mortality as well as hospitalization in patients with severe systolic HF [12]. Additionally, PMVR results in significant improvement of figural memory and planning ability as well as psychosocial functioning in HF patients [13]. Since there is a risk of withholding optimal medical treatment to very sick HF patients and also a discussion about patients being “too sick” for PMVR, we aimed to assess the effects of PMVR using the MitraClip on psychological and cognitive functioning as compared with the initial severity of psychological- and cognitive malfunctioning prior to PMVR.

## Materials and methods

In a pre–post-interventional controlled trial, 40 patients with end-stage HF and severe mitral valve regurgitation were assessed pre- (1 day before; *t*0) and post-(6 weeks after; *t*1) MC intervention (MC; *n *= 40). We choose a 6-week observation period, since there is evidence that patients recover from the minimal-invasive MC procedure within 4 weeks [14] and to exclude unrelated effects which might occur within a longer period of time. The sample consisted of patients referred to the outpatient setting of the Heart Failure Section of the Department of Cardiology, Angiology and Pneumology of the Medical Hospital, University of Heidelberg between 2014 and 2016 where all examinations including the MC procedure and cognitive testing were carried out. General inclusion criteria for the MC intervention were suffering from ischemic or dilated cardiomyopathy showing an ejection fraction of 35% or lower or being categorized as high-risk surgical HF patients due to reduced systolic function of below 45% combined with a Society of Thoracic Surgeons’ (STS) score of > 8%. The main exclusion criteria were morphological properties of the MV that would make MitraClip implantation unlikely or impossible as published previously by Feldman et al. [14]. All patients received stable optimized individual target heart failure medication at least for 3 months prior to MC intervention. We assessed questionnaires on socio-demographic data (age, sex, school level), verbal- and education-related intelligence, eye vision, and handedness [15], followed by the participants’ psychometric and neuropsychological assessment. Total testing time took approximately 150 min for both pre-assessment (*t*0) and re-assessment (*t*1). All participants were asked to avoid eating and drinking caffeinated beverages for 1 h before and smoking nicotine or consuming alcohol for 24 h prior to assessment. For cognitive testing, general inclusion criteria were right-handedness, normal or corrected-to-normal vision, and native German language. Written informed consent was obtained from all participants as approved by the local ethics committee of the University of Heidelberg (No. S-243/2013).

The Cognitive Basic Assessment test set (COGBAT) from the Vienna test system [16] efficiently assesses the key neuropsychological dimensions of attention, memory, executive functions, and processing speed and has been validated across neurological and psychiatric populations [17, 18]. The tests are presented in a fixed order and take 60 min to complete. With regard to reliability, internal consistencies expressed as Cronbach’s α = 0.70–0.92 can be determined as adequate to excellent. The assessed COGBAT cognitive dimensions are described in detail in our previous research [13]. Moreover, the COGBAT is cross-test normed enabling the comparison of individual scores to representative sample scores for the following age groups (in years): ‘16 to 30’ (*N *= 127), ‘31 to 50’ (*N *= 151), and ‘51 to 80’ (*N *= 141).

We assessed the Patient Health Questionnaire (PHQ-D; German version [19]), which is used to diagnose common mental health disorders. For the purpose of this study, the scores of the 9-item depression module are reported (PHQ-9; [20]). The PHQ-9 depression module is designed to assess depressive symptoms, disorder severity, and symptom development [21]. It shows very good validity [22, 23] as well as sensitivity for change [24] measures and has yielded good results in testing depression in patients with chronic somatic diseases [25]. Mental and physical health-related quality of life was measured using the 36-item Short-Form General Health Survey (SF-36; German version [26]), which shows good reliability and internal consistency, and satisfying discriminant and convergent validity [26]. In this paper, the term psychosocial functioning refers to the degree of depression (i.e., PHQ-9) as well as to the quality of life (i.e., SF-36).

To control for verbal and education-related intelligence, participants completed the Multiple Selection Vocabulary Test (“Mehrfachwahl-Wortschatz-Intelligenztest”; MWT-B; [27]; see Table 1) at *t*0. The MWT-B comprises 37 rows of words, each of which consists of one German word which is colloquially, scientifically, or eruditely familiar and four nonsense words. Participants are required to recognize familiar words that exist in the German language. Scores are based on the number of correctly identified words (maximum of 37). Investigations on re-test reliability have revealed high correlations [28].

**Table 1:** Sample description for assessed MitraClip (MC group; *N *= 40)

	Pre (*t*0)	Post (*t*1)
COGBAT norm percentile (≥ 50 = healthy)		
Planning ability (median; IQR)	30 (11.25 to 42.50)	65 (35 to 80)
Figural memory (median; IQR)	22.5 (0 to 50)	45 (16.25 to 75)
Patient characteristics and biomarkers		
Age (years; median)	73	–
Sex (female)	19/40 (47.5%)	–
MWT-B intelligence quotient (IQ)	110.83 ± 16.3	–
STS-score (%)	5.16 (5.24)	–
Psychopharmacological medication (*n*)	6 (15%)	–
Ejection fraction (%)	35 ± 15	35 ± 16
6 min walk distance test (m)	366 ± 7	415 ± 115
NT-ProBNP (ng/L) (median)	3880	3627
hsTnT (pg/mL)	36 ± 33	46 ± 80
Pathogenesis and comorbidity		
ICMP (%)	21/40 (52.5%)	–
DCMP (%)	19/40 (47.5%)	–
Implantable cardioverter defibrillator (%)	15/40 (37.5%)	–
Prior cardiothoracic surgery (%)	9/40 (22.5%)	–
Atrial fibrillation (%)	20/40 (50%)	–
Prior stroke (%)	4/40 (10%)	–
Increased retention values (> 1.3 mg/dL) (%)	11/40 (27.5%)	–
Severe sleep apnea syndrome (%)	4/40 (10%)	–
Diabetes mellitus (%)	6/40 (15%)	–
Pulmonary disease	8/40 (20%)	–
Cancer (%)	1/40 (2.5%)	–
Functional Mitral valve regurgitation (FMR)		
FMR ≤ I°	0	14/40 (35%)
FMR ≤ II°	0	23/40 (57.5%)
FMR > II°–< III°	4/40 (10%)	3/40 (7.5%)
FMR III°	11/40 (27.5%)	0
FMR IV°	23/40 (57.5%)	0
NYHA functional classification		
NYHA I	0	1/40 (2.5%)
NYHA II	3/40 (7.5%)	24/40 (60%)
NYHA III	32/40 (80%)	15/40 (37.5%)
NYHA IV	5/40 (12.5%)	0

*MWT-B* multiple selection vocabulary test, *STS-score* risk score of the Society of Thoracic Surgeons to predict operative mortality of adult cardiac surgery, *NT-ProBNP* N-terminal pro-brain natriuretic peptide, *hsTnT* high-sensitive Troponin T, *ICMP* ischemic cardiomyopathy, *DCMP* dilated cardiomyopathy, *NYHA* New York Heart Association, *SD* standard deviation, *IQR* interquartile range

A detailed overview of clinical, echocardiography, and invasive haemodynamic data as well as comorbidities is summarized in Table 1.

MC percutaneous edge-to-edge mitral valve repair procedure was performed under general anesthesia and under transesophageal echocardiographic guidance. Quantification of mitral regurgitation was carried out using transthoracic echocardiography, transesophageal echocardiography, left ventricular angiogram and invasive measurements of pulmonary artery pressure and left atrial pressure as described previously [29]. After atrial transseptal puncture, mitral leaflets were approximated using the 24-French MitraClip^®^ device (Abbott Vascular, Santa Clara, CA, USA). In the case of insufficient mitral regurgitation reduction with a single device, the device may either be removed or a second device placed [14, 29]. Hemostasis was achieved using the ProGlide^®^ device (Abbott Vascular, Santa Clara, CA, USA) as previously described by Geis et al. [30]. After the procedure, patients were transferred to the intensive care unit for at least 24 h.

Data were analyzed using SPSS (Version 25; SPSS Inc., Chicago, IL, USA). First, dependent t tests (*t*0 vs. *t*1) were conducted to reveal improvements of cognitive performance in the MC group. A *p* value < 0.05 (two tailed) was considered statistically significant. Second, to determine predictors of the detected cognitive performance improvements, bivariate linear regressions were calculated (MC group; *N *= 40) with cognitive performance improvements (Δ*T*: *t*1–*t*0) as dependent variable and all key variables pre-intervention (*t*0) as independent variables. To minimize Type I errors, a Bonferroni familywise *α* error correction was applied on one-tailed *p* values (6-test family; corrected threshold *p* ≤ 0.008). Furthermore, repeated measures MANOVAs were conducted to explore possible group differences for patients with ischemic vs. dilated cardiomyopathy (ICMP vs. DCMP) of figural memory, planning ability, and cognitive flexibility with the within-subject factor ‘Time’ (*t*0 vs. *t*1).

## Results

### Participant baseline data

Table 1 shows participants’ baseline data. The MC patient group ranged from 31 to 86 years (*M *= 69.78, *SD *= 11.3), and 47.5% were female. With regard to the underlying pathogenesis of the existing HF, 52.5% of our patients (*n *= 21) show HF due to ischemic cardiomyopathy (ICMP) and 47.5% of our patients (*n *= 19) suffered from dilated cardiomyopathy (DCMP). At the time of assessment, six of the 40 HF patients received psychopharmacological or opioid medication (antidepressants *n* = 3, benzodiazepines *n* = 2; opioid analgesics *n* = 1).

### Comparison of cognitive functioning before and after the MitraClip intervention

**Fig. 1:**
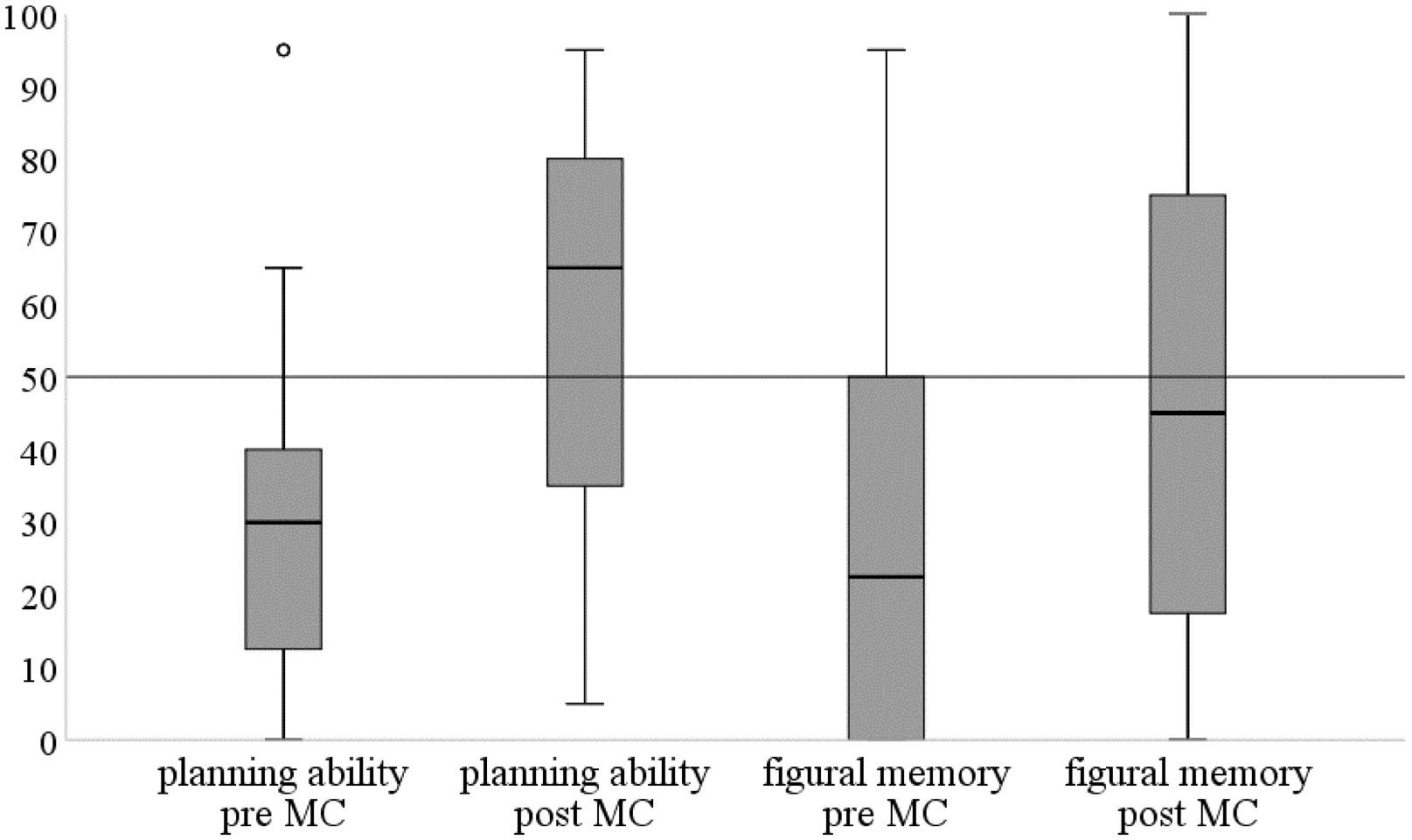
Individual’s results in pre–post-MitraClip planning ability as well as figural memory (MC group; *n* = 40) compared to the normative COGBAT sample, while test scores ≥ 50 indicate normal/ healthy cognitive functioning and test scores < 50 indicate impaired cognitive functioning in the respective domain. For planning ability, median test score values are shifting in the healthy area at t1 (post-MC)

As depicted in Table 2, dependent *t* tests revealed a significant improvement of the MC group (*N *= 40) for figural memory (learning sum: *t*(39) = − 7.29, *p* < 0.001, *d* = 1.15), executive functions based on planning ability (*t*(39) = − 4.83, *p* < 0.001, *d* = 0.76) as well as cognitive flexibility (TMT B: *t*(39) = 3.82, *p* < 0.001, *d* = 0.60) from *t*0 to *t*1. To compare test results of the improved cognitive functioning in the MC group to a sample of age-matched healthy controls, the individual’s results were compared to the representative COGBAT sample including healthy persons over all age groups (see Table 3 and Fig. 1). For ‘planning ability’, 78% (*t*0) versus 41.5% (*t*1), and for ‘figural memory’, 73.2% (*t*0) versus 58.5% (*t*1) obtained test scores below the 45th percentile compared to the COGBAT normative sample; this means that cognitive performance was below the normal range before the MC intervention and that particularly planning ability improved showing test scores within the normal range post-MC intervention (median = 65).

**Table 2:** Differences for psychosocial, neuropsychological, and clinical assessment pre (*t*0) and after 6 weeks (*t*1) post-MitraClip intervention

	MC group (*N *= 40) Mean (SD)		*t*	*p*
	*t*0	*t*1		
Psychosocial functioning^a^				
Depression-score (PHQ-9; 0–27)	6.44 (4.45)	3.78 (3.15)	4.57	< .001
Psychological well-being (SF-36; 0–100)	68.75 (22.50)	77.88 (15.57)	− 2.83	0.008
Physical well-being (SF-36; 0–100)	43.66 (25.02)	59.84 (26.79)	− 3.81	0.001
Cognitive functioning				
Figural memory learning sum (FGT; 0–45)	18.23 (8.98)	24.55 (8.34)	− 7.29	< .001
Planning ability (TOL; 0–24)	11.75 (4.14)	15.25 (3.36)	− 4.83	< .001
Cognitive flexibility (TMT: Part B) [s]	120.15 (63.54)	99.35 (47.83)	3.82	< .001
Clinical parameters				
LVEF	34.60 (14.95)	34.57 (15.64)	0.03	n.s.
6-MWT [s]	365.69 (6.64)	414.52 (115.40)	− 3.57	0.001
FMR	2.83 (0.64)	1.35 (0.48)	12.43	< .001
NYHA	3.05 (0.54)	2.34 (.49)	8.14	< .001
NT-ProBNP	10,161.9 (20,141.1)	8159.5 (16,704.3)	2.28	0.029

*PHQ-9* Patient Health Questionnaire, *SF-36* Short-Form Health Survey, *FGT* figural memory test, *TOL*, Tower of London–Freiburg Version, *TMT-L* trail making test–Langensteinbach version, *LVEF* left ventricular ejection fraction, *6-MWT* 6-minute walk test, *FMR* functional mitral valve regurgitation, *NT-ProBNP* N-terminal pro-brain natriuretic peptide; Values are presented means, standard deviations, *t* value and *p* value. ^a^Psychosocial assessment for *N *= 32

**Table 3:** Individual’s results in pre–post-MitraClip planning ability as well as figural memory (MC group; *N *= 40) compared to the normative COGBAT sample

	Percentile (*t*0)				Percentile (*t*1)			
	≤ 20	≤ 35	≤ 50	> 50	≤ 20	≤ 35	≤ 50	> 50
Planning ability, *n* (%)	17 (42.5)	30 (75.0)	31 (77.5)	9 (22.5)	6 (15.0)	12 (30.0)	16 (40.0)	24 (60.0)
Figural memory, *n* (%)	20 (50.0)	29 (72.5)	31 (77.5)	9 (22.5)	11 (27.5)	19 (47.5)	23 (57.5)	17 (42.5)

### Predictors of change in cognitive functioning due to the MitraClip intervention

Table 4 depicts bivariate linear regressions of baseline scores (*t*0) in (i) cognitive functioning (i.e., planning ability, figural learning, cognitive flexibility), and (ii) psychosocial functioning (i.e., PHQ-9, SF-36 psychological well-being, SF-36 physical well-being) and age with cognitive performance improvements (*t*1–*t*0) due to the MC intervention. While psychosocial functioning and age were not associated with improvements in cognitive functioning, impaired planning ability, figural memory as well as cognitive flexibility at *t*0 were associated with better cognitive functioning. Cognitive performance was not controlled for depression, as no correlations were found for pre–post-changes in PHQ-9 scores and pre–post-changes in memory and executive function (all *p* > 0.05).

**Table 4:** Bivariate regressions (MC group; *N *= 40) of cognitive performance improvement with psychological- and cognitive malfunctioning, and age before PMVR

*t*0	Planning ability (*t*1–*t*0)		Figural memory (*t*1–*t*0)		Cognitive flexibility (*t*1–*t*0)	
	*B*	95% CI	*B*	95% CI	*B*	95% CI
TOL	− 0.78	[− 1.04, − 0.53]	− 0.26	[− 0.69, 0.17]	1.88	[− 0.78, 4.53]
FGT	− 0.25	[− 0.39, − 0.10]	− 0.26	[− 0.44, − 0.07]	1.08	[− 0.13, 2.29]
TMT B	0.03	[0.01, 0.05]	0.01	[− 0.01, 0.04]	− 0.36	[− 0.50, − 0.23]
PHQ-9^a^	0.19	[− 0.23, 0.60]	0.12	[− 0.29, 0.52]	1.21	[− 1.83, 4.24]
SF36 mental^a^	− 0.04	[− 0.37, 0.30]	− 0.12	[− 0.44, 0.20]	0.06	[− 0.55, 0.67]
SF36 physical^a^	− 0.17	[− 0.54, 0.20]	− 0.01	[− 0.36, 0.38]	0.01	[− 0.55, 0.56]
Age	0.10	[− 0.03, 0.23]	− 0.05	[− 0.21, 0.11]	− 0.29	[− 1.28, 0.71]

*TOL* Tower of London–Freiburg Version, *FGT* figural memory test, *TMT B* trail making test part B, *PHQ-9* Patient Health Questionnaire, *SF-36* Short-Form Health Survey, *SF36 mental* psychological well-being, *SF36 physical* physical well-being. ^a^*N *= 32 completed psychological assessment both at *t*0 and at *t*1

### Comparison of psychological assessment before and after the MitraClip intervention

Table 3 depicts MC patients’ results of psychological assessment. Out of *N *= 40 MC patients, only *N *= 32 at *t*0 and *N *= 37 at *t*1 completed psychological assessment. Dependent *t*-tests (*t*0 vs. *t*1) were conducted, showing significantly improved depression scores (PHQ-9: *t*(31) = 4.57, *p *< 0.001, *d* = 0.81) as well as quality of life (SF36 psychological well-being: *t*(31) = − 2.83, *p *= 0.01, *d* = 0.5; SF36 physical well-being: *t*(31) = − 3.81, *p *< 0.001, *d* = 0.68) scores post-MC.

### Cognitive performance before and after MitraClip intervention: ICMP vs. DCMP

Repeated MANOVAs were separately conducted for various cognitive domains with the between-subject factor ‘Group’ (ICMP; *n* = 21 vs. DCMP; *n* = 19) and the within-subject factor ‘Time’ (*t*0 vs. *t*1). There were no significant effects.

## Discussion

The present study examined cognitive functioning in HF patients pre- and post-MC intervention. In line with our previous results [13], figural memory and executive functioning were significantly increased due to percutaneous mitral valve repair (PMVR) using MitraClip in HF patients.

Our results showed that HF patients show additional improvement in cognitive flexibility, reflected by the improved TMT B test scores 6 weeks after PMVR. Most importantly, the evaluation of cognitive functioning predictors revealed that poorer baseline figural memory and executive functioning correlated significantly with greater cognitive performance improvements, in the respective domains following the MC intervention irrespective of medical and psychological covariates.

The pathophysiology of impaired cognitive functioning seems to be diverse in elderly HF patients as reflected by their regular decline in decreased cognitive functioning ‘per se’ [31, 32] due to age-related changes in the brain [33–35]. Reduced cognitive reserve in the elderly is linked to a general higher risk of cognitive impairment [36]. HF is an additional risk factor for various disorders like dementia [37] or multiple cerebral emboli [38, 39]. In addition, post-operative cognitive dysfunction affects up to 54% of patients shortly after cardiac surgery [40]. Therefore, one could assume that elderly HF patients with poorer cognitive ability may fail to benefit from the MC intervention at a cognitive level and are particularly vulnerable to cognitive impairment after the intervention [41]. However, in our sample age did *not* correlate with baseline scores and cognitive outcome post-MC intervention. In contrast, our results suggest that the age of HF patients plays a minor role, yet HF patients with poorer baseline cognitive functioning benefit the *most* with regard to their cognitive performance post-MC intervention.

One study using magnetic resonance imaging in 13 high-risk patients undergoing percutaneous mitral valve reconstruction reported that the MitraClip procedure resulted in newly acquired cerebral lesions in 9 out of 13 patients [42]. Similarly, others reported peri-interventional thrombus formation and stroke [43–45]. However, in large registries the incidence of clinical relevant stroke within 30 days after MC is low and varies between 0.7 and 2.6% [46–49]. Furthermore, we recently published a very low rate (0.2%; 1/470 patients) of 30-days peri-interventional stroke in patients treated in our hospital due to temporary oral anticoagulation for 30 days using coumadin [50]. Therefore, we might speculate that avoiding peri-interventional stroke due to temporary oral anticoagulation using Coumadin for 30 days might support our findings of improved cognitive function.

The perfusion hypothesis states that insufficient blood circulation underlies cognitive deficits in patients with chronic heart failure [13]. Our patients suffered from significant systolic heart failure. Efficacy of the left ventricular (LV) function is further compromised in our patients due to severe mitral valve regurgitation. Due to mitral valve repair, LV volume overload is reduced and, thus, the efficacy of LV function can be improved. Systolic LVEF remains unaltered, which is expected and in line with previous results [29]. Increased cognitive performance appears due to the reduction of mitral regurgitation but not due to improvement of heart failure. With regard to the underlying pathogenesis of HF, our results revealed that patients with ICMP vs. DCMP equally improved their cognitive as well as mental functioning.

Another important issue is whether the observed effects on cognitive function might be a consequence of different medical therapy post-MC intervention. With regard to this aspect, all patients received stable optimized individual target heart failure medication at least for 3 months prior to MC intervention. As previously published, no significant alteration in HF medication (regarding beta-blockers, ACE inhibitors/angiotensin receptor blockers and aldosterone receptor inhibitors) was observed within a 12-month post-MC observation period [29]. Thus, improvement of cognitive function is likely to be independent of medication in our study sample.

In a meta-analysis, McDermott et al. [51] found significant correlations between depression severity and the cognitive domains executive function, processing speed and episodic memory. Likewise, an association between depression symptoms, assessed by the Beck Depression Inventory II, and impaired executive functioning, measured by the Frontal Assessment Battery, has been shown for older patients with HF [52]. Hence, the examination of cognitive performance in HF patients requires careful consideration of psychological well-being, particularly with respect to the assessment of depression symptoms. Most importantly, psychosocial functioning (i.e., depression and psychological/physical well-being) *did neither* predict baseline cognitive functioning nor cognitive performance outcomes in the MC group, which is in line with previous results [53], since depressive symptoms in the current study are “sub-clinical” and considered as a simple somatically caused side effect of HF.

## Limitations

A shortcoming of the present study is the small sample size which might have limited power to detect significant effects. In future studies, additional measurement times and longer intervals between assessments might be beneficial in the investigation of the MC intervention’s long-term effects on both psychosocial and cognitive parameters.

## Conclusions

Our results show that (1) percutaneous mitral valve repair using the MitraClip is an efficient tool to improve both cognitive and psychological outcomes which might be caused by haemodynamic changes in HF patients irrespective of the pathogenesis of HF. Second (2) HF patients with impaired baseline scores in cognitive performance benefited most from the MitraClip procedure, showing improved executive function with post-intervention scores within the normal range. Lastly, (3) after careful interdisciplinary discussion, the MitraClip procedure should not be withheld from elderly HF patients—despite the evidence of reduced cognitive reserve.

## Abbreviations

HF: systolic heart failure
6-MWT: 6-min walk test
COGBAT: Cognitive Basic Assessment
DCMP: dilated cardiomyopathy
FGT: figural memory test
FMR: functional Mitral valve regurgitation
HF: heart failure
hsTnT: high-sensitive Troponin T
ICMP: ischemic cardiomyopathy
LVEF: left ventricular ejection fraction
MC: MitraClip
MR: mitral valve regurgitation
MWT-B: Multiple selection vocabulary test: verbal and education-related intelligence
NT-ProBNP: N-terminal pro-brain natriuretic peptide
NYHA: New York Heart Association
PHQ: Patient Health Questionnaire
PMVR: Percutaneous mitral valve repair
SF36: Short-Form Health Survey
STS: risk score of the Society of Thoracic Surgeons to predict operative mortality of adult cardiac surgery
TMT: trail making test
TOL: Tower of London: executive function—planning ability

## References

[1] Kasper D, et al. i sur., ur. Harrison’s principles of internal medicine. 19. izd. 2015, New York: McGraw Hill.

[2] Longo DL, et al. Harrison’s principles of internal medicine 18E Vol 2 EB. 2012: McGraw Hill Professional.

[3] Goliasch G (2018). Refining the prognostic impact of functional mitral regurgitation in chronic heart failure. Eur Heart J.

[4] Bauer L (2012). A brief neuropsychological battery for use in the chronic heart failure population. Eur J Cardiovasc Nurs.

[5] Pressler SJ (2010). Cognitive deficits in chronic heart failure. Nurs Res.

[6] Askoxylakis V (2010). Long-term survival of cancer patients compared to heart failure and stroke: a systematic review. BMC Cancer.

[7] Bennett SJ, Sauve MJ (2003). Cognitive deficits in patients with heart failure: a review of the literature. J Cardiovasc Nurs.

[8] Bui AL, Horwich TB, Fonarow GC (2011). Epidemiology and risk profile of heart failure. Nat Rev Cardiol.

[9] Mapelli D (2011). Neuropsychological profile in a large group of heart transplant candidates. PLoS ONE.

[10] Peters-Klimm F (2012). Physician and patient predictors of evidence-based prescribing in heart failure: a multilevel study. PLoS ONE.

[11] Sila CA (2007). Cognitive impairment in chronic heart failure. Cleve Clin J Med.

[12] Stone GW (2018). Transcatheter mitral-valve repair in patients with heart failure. N Engl J Med.

[13] Nikendei C (2016). The effects of mitral valve repair on memory performance, executive function, and psychological measures in patients with heart failure. Psychosom Med.

[14] Feldman T (2011). Percutaneous repair or surgery for mitral regurgitation. N Engl J Med.

[15] Oldfield RC (1971). The assessment and analysis of handedness: the Edinburgh inventory. Neuropsychologia.

[16] Aschenbrenner S (2012). Testset COGBAT.

[17] Schwert C (2018). Biased neurocognitive self-perception in depressive and in healthy persons. J Affect Disord.

[18] Sharma A (2017). Relationship between serum calcium and neuropsychological performance might indicate etiological heterogeneity underlying cognitive deficits in schizophrenia and depression. Psychiatry Res.

[19] Lowe B (2010). A 4-item measure of depression and anxiety: validation and standardization of the Patient Health Questionnaire-4 (PHQ-4) in the general population. J Affect Disord.

[20] Kroenke K, Spitzer RL (2002). The PHQ-9: a new depression diagnostic and severity measure. Psychiatr Ann.

[21] Lowe B, Kroenke K, Grafe K (2005). Detecting and monitoring depression with a two-item questionnaire (PHQ-2). J Psychosom Res.

[22] Lowe B (2004). Diagnosing ICD-10 depressive episodes: superior criterion validity of the Patient Health Questionnaire. Psychother Psychosom.

[23] Lowe B (2004). Comparative validity of three screening questionnaires for DSM-IV depressive disorders and physicians’ diagnoses. J Affect Disord.

[24] Lowe B (2004). Measuring depression outcome with a brief self-report instrument: sensitivity to change of the Patient Health Questionnaire (PHQ-9). J Affect Disord.

[25] Nikendei C (2018). Depression profile in cancer patients and patients without a chronic somatic disease. Psychooncology.

[26] Bullinger M, Kirchberger I. Der SF-36 Fragebogen zum Gesundheitszustand. (SF-36)-Handbuch für die deutschsprachige Fragebogenversion. Hogrefe, Göttingen, 1998.

[27] Lehrl S. Mehrfachwahl-Wortschatz-Intelligenztest: MWT-B [Multiple Choice Vocabulary Test, version B]. 2005, Balingen, Germany: apitta.

[28] Lienert G. Testaufbau und Testanalyse. München Lienert, G.: Testaufbau und Testanalyse. 1989, München–Weinheim.

[29] Pleger ST (2013). One year clinical efficacy and reverse cardiac remodelling in patients with severe mitral regurgitation and reduced ejection fraction after MitraClip implantation. Eur J Heart Fail.

[30] Geis NA (2015). Feasibility and clinical benefit of a suture-mediated closure device for femoral vein access after percutaneous edge-to-edge mitral valve repair. EuroIntervention.

[31] McCurry SM (2001). Neuropsychological test performance in a cognitively intact sample of older Japanese American adults. Arch Clin Neuropsychol.

[32] Royall DR (2005). Normal rates of cognitive change in successful aging: the freedom house study. J Int Neuropsychol Soc.

[33] de Leeuw FE (2001). Prevalence of cerebral white matter lesions in elderly people: a population based magnetic resonance imaging study. The Rotterdam Scan Study. J Neurol Neurosurg Psychiatry.

[34] Oosterman JM (2010). Assessing mental flexibility: neuroanatomical and neuropsychological correlates of the Trail Making Test in elderly people. Clin Neuropsychol.

[35] Raz N (2004). Differential aging of the medial temporal lobe: a study of a five-year change. Neurology.

[36] Almkvist O, Tallberg IM (2009). Cognitive decline from estimated premorbid status predicts neurodegeneration in Alzheimer’s disease. Neuropsychology.

[37] Qiu C (2006). Heart failure and risk of dementia and Alzheimer disease: a population-based cohort study. Arch Intern Med.

[38] Justin BN, Turek M, Hakim AM (2013). Heart disease as a risk factor for dementia. Clin Epidemiol.

[39] Picano E (2014). Cognitive impairment and cardiovascular disease: so near, so far. Int J Cardiol.

[40] Androsova G (2015). Biomarkers of postoperative delirium and cognitive dysfunction. Front Aging Neurosci.

[41] Schall RR (1989). Cognitive function in patients with symptomatic dilated cardiomyopathy before and after cardiac transplantation. J Am Coll Cardiol.

[42] Barth S (2017). Incidence and clinical impact of cerebral lesions after the MitraClipA(R) procedure. J Heart Valve Dis.

[43] Bekeredjian R (2011). Large atrial thrombus formation after MitraClip implantation: is anticoagulation mandatory?. J Heart Valve Dis.

[44] Hamm K (2013). Stroke and thrombus formation appending to the MitraClip: what is the appropriate anticoagulation regimen?. J Heart Valve Dis.

[45] Frerker C (2016). Cerebral protection during MitraClip implantation: initial experience at 2 centers. JACC Cardiovasc Interv.

[46] Sorajja P (2016). Initial experience with commercial transcatheter mitral valve repair in the united states. J Am Coll Cardiol.

[47] Maisano F (2013). Percutaneous mitral valve interventions in the real world: early and 1-year results from the ACCESS-EU, a prospective, multicenter, nonrandomized post-approval study of the MitraClip therapy in Europe. J Am Coll Cardiol.

[48] Whitlow PL (2012). Acute and 12-month results with catheter-based mitral valve leaflet repair: the EVEREST II (Endovascular Valve Edge-to-Edge Repair) High Risk Study. J Am Coll Cardiol.

[49] Glower DD (2014). Percutaneous mitral valve repair for mitral regurgitation in high-risk patients: results of the EVEREST II study. J Am Coll Cardiol.

[50] Geis N (2019). Temporary oral anticoagulation after MitraClip—a strategy to lower the incidence of post-procedural stroke?. Acta Cardiol.

[51] McDermott LM, Ebmeier KP (2009). A meta-analysis of depression severity and cognitive function. J Affect Disord.

[52] Alosco ML (2013). The interactive effects of cerebral perfusion and depression on cognitive function in older adults with heart failure. Psychosom Med.

[53] Steinberg G (2011). Peak oxygen uptake and left ventricular ejection fraction, but not depressive symptoms, are associated with cognitive impairment in patients with chronic heart failure. Int J Gen Med.

